# Factors Influencing the Steady-State Plasma Concentration of Imatinib Mesylate in Patients With Gastrointestinal Stromal Tumors and Chronic Myeloid Leukemia

**DOI:** 10.3389/fphar.2020.569843

**Published:** 2020-11-25

**Authors:** Yan Chen, Xiuhua Dong, QiuJu Wang, ZhiXi Liu, XinWei Dong, Sanjun Shi, HongTao Xiao

**Affiliations:** ^1^ Department of Clinical Pharmacy, Sichuan Cancer Hospital and Institute, Sichuan Cancer Center, School of Medicine, University of Electronic Science and Technology of China, Chengdu, China; ^2^ Department of Stomatology, The 1st Affiliated Hospital of Chengdu Medical College, Chengdu, China; ^3^ Department of Clinical Laboratory, Sichuan Cancer Hospital and Institute, Sichuan Cancer Center, School of Medicine, University of Electronic Science and Technology of China, Chengdu, China; ^4^ Department of Pharmacy, Chengdu Medical College, Chengdu, China; ^5^ School of Pharmacy, Chengdu University of Traditional Chinese Medicine, Chengdu, China; ^6^ Personalized Drug Therapy Key Laboratory of Sichuan Province, Chengdu, China

**Keywords:** imatinib, therapeutic drug monitoring, influence factors, gastrointestinal stromal tumor, chronic myeloid leukemia

## Abstract

Imatinib mesylate (IM) is the standard treatment for advanced, metastatic gastrointestinal stromal tumors (GISTs) and chronic myeloid leukemia (CML) with a fixed daily standard dosage via the oral route. Interindividual and intraindividual variability in plasma concentrations have been closely linked to the efficacy of IM therapy. Therefore, this review identifies and describes the key factors influencing the plasma concentration of IM in patients with GISTs and CML. We used the following keywords to search the PubMed, EMBASE, Ovid, Wangfang, and CNKI databases to identify published reports: IM, plasma concentration, GISTs, CML, drug combination/interaction, pathology, and genotype/genetic polymorphism, either alone or in combination. This literature review revealed that only 10 countries have reported the mean concentrations of IM in GISTs or CML patients and the clinical outcomes in different ethnic groups and populations. There were totally 24 different gene polymorphisms, which were examined for any potential influence on the steady-state plasma concentration of IM. As a result, some genotype locus made discrepant conclusion. Herein, the more sample capacity, multicenter, long-term study was worthy to carry out. Eleven reports were enumerated on clinical drug interactions with IM, while there is not sufficient information on the pharmacokinetic parameters altered by drug combinations with IM that could help in investigating the actual drug interactions. The drug interaction with IM should be paid more attention in the future research.

## Introduction

For the last 15 years, imatinib mesylate (IM) has been the first line of treatment for advanced, metastatic gastrointestinal stromal tumors (GISTs) (Rutkowski et al., 2020) and has also been approved as therapy for Philadelphia chromosome-positive chronic myeloid leukemia (CML) (Markovic et al., 2020) via the oral route (Banegas et al., 2019). IM has dramatically improved the quality of life and long-term prognosis and increased the overall survival rates of patients with GISTs or CML (Salem et al., 2019). IM is an important example of oral, targeted cancer therapy. However, on the one hand, 1 in 2–4 patients with GISTs still exhibits a low response to IM because of intolerance or adverse effects (Bednarski et al., 2014). On the other hand, nearly 10–15% of early chronic stage patients tend to display primary or acquired cytogenetic resistance, which hinders the overall success of IM therapy (Onmaz et al., 2019).

Interpatient differences in therapeutic response may occur partly because of pharmacokinetic (PK) variability, which has been estimated to be ∼60 and 71% in IM steady-state trough concentrations in patients with GISTs or CML, respectively (Gandia et al., 2013; Zhuang et al., 2018). PK variability, which is mainly caused by physiological, pathological, genetic, demographic, and environmental factors, manifests as a broad range of plasma trough levels (C_min_) in patients receiving the same dosage, usually 400 mg/d of IM (Widmer et al., 2014). The main side effects of IM in patients with GISTs, such as myelosuppression and periocular edema, have been observed in ∼50% of the patients (Ondecker et al., 2018). Although most of these side effects have been classified as moderate, grade-1, or grade-2 adverse events, according to the Common Terminology Criteria for Adverse Events, the quality of life could be adversely affected to a significant degree by physical and psychosocial discomfort (Abu-Amna et al., 2016). Therapeutic drug monitoring (TDM) has been recommended in IM therapy as fixed doses of IM are usually administered for a prolonged period (Vithanachchi et al., 2020). What’s more, TDM for IM has been playing an equivalently important role in drug response and improving the efficacy and safety of treatments. Plasma concentrations must be considered to determine whether a target drug achieves a complete cytogenetic response (CCyR) or a major molecular response (MMR) at different time points (Groenland et al., 2019). Consequently, with the help of TDM clinicians could minimize the risk of major adverse reactions and optimize therapy through the individualization of dosage to get satisfactory results (Cardoso et al., 2018). Currently, a latest review (Buclin et al., 2020) summarized some standard steps of TDM for some specific drug using IM as the example, that is, i) judging whether IM is a candidate to TDM, ii) thinking about the normal range for plasma concentration of IM, iii) defining what is the effective target for the concentration of IM, iv) reflecting on how to adjust the dosage of IM close to target concentration, and v) summarizing evidence supporting the usefulness of TDM for IM. This work made great contribution for explanation of the importance of monitoring with IM and listed the standard process in monitoring IM. However, the factors influencing the plasma concentration of IM in clinic should still be considered and discussed in depth when the clinician decided to monitor IM.

To date, IM is recommended for TDM according to the guideline of National Comprehensive Cancer Network (NCCN 2020 edition) (Alessandrino et al., 2019). Based on the literature research, there is only one report, which defined the specific range of plasma concentration of IM, that pointed out that C_min_ of IM ranging from 1,000 to 3,180 μg/L was verified effective to the patients with CML (Guilhot et al., 2012). When the C_min_ level was above 3,180 μg/L, it was associated with a higher frequency of some grade 3/4 adverse events. Nevertheless, most of IM-related plasma monitoring researches only recognized the threshold of 1,000 μg/L (Smy et al., 2020).

**FIGURE 1:**
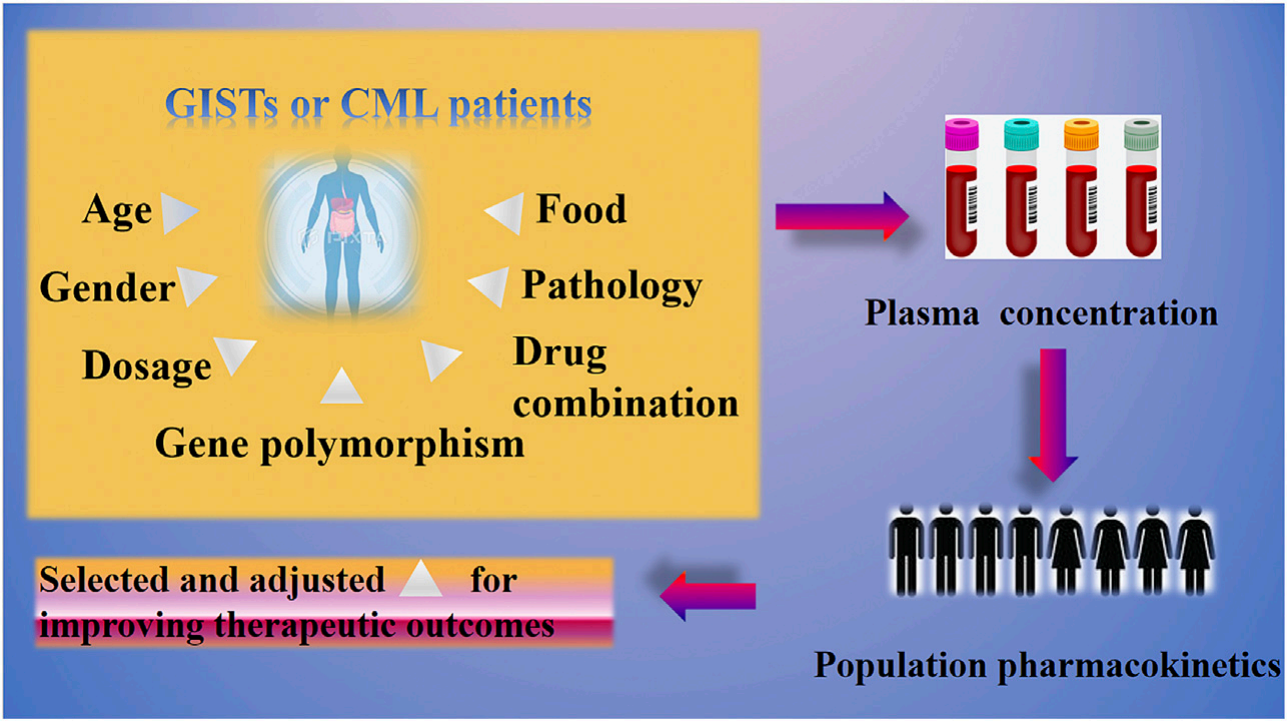
Graphical abstract.

Due to this “huge effective range” of IM plasma concentration, we want to know which key factors affected this outcome. We believe that a deeper understanding of the influencing factors could bring easier treatment of GISTs and CML based on the regimen of IM. Herein, the aim of this review was to discuss the key elements that influence steady-state plasma concentrations of IM in different patient groups to facilitate rational use of IM in clinical settings. The graphical abstract is shown in Figure 1. We shall summarize and discuss this from the aspects of age, gender, dosage, genetic polymorphism, drug combination, pathology, and food to analyze the potential impact for IM in clinic use.

## Methods

### Data Sources

We searched PubMed, EMBASE, and Ovid databases to identify published reports with keywords such as IM, plasma concentration, GISTs, CML, drug combination/interaction, pathology, and genotype/genetic polymorphism, either alone or in combination. We also used the aforementioned keywords to search the CNKI and Wangfang databases for reports up to December 2019 in the Chinese language. The search was completed on a single day to complete and to simplify the study design.

### Data Analysis

We used Microsoft Excel 2010 software (Microsoft, America) to record and sort the following information: title, publication year, author, country, type of study (basic research, clinical research, or review), number of patients, dosage of IM, genetic polymorphism in the study, and major results of the study. If a report lacked information about steady-state plasma concentrations of IM, we searched for these “missing” data and entered the information into the datasheet.

## Results and Discussion

### Current Usage of Imatinib Mesylate for Gastrointestinal Stromal Tumors and Chronic Myeloid Leukemia

IM is a well-known, orally administered tyrosine kinase inhibitor (TKI) (Musumeci et al., 2015). The bioavailability of IM is ∼90% via the oral route, as it is metabolized predominantly by cytochrome P450 enzymes (Musumeci et al., 2015), CYP3A4 (Maddin et al., 2016), and CYP3A5 (Cargnin et al., 2018), to its major circulating active metabolite, *N*-desmethyl imatinib (NDI)/CGP74588 (Lin et al., 2019). This metabolite also binds to the ATP binding pocket of the tyrosine kinase domain (Josephs et al., 2013). The terminal half-life of IM is ∼18 h, whereas the half-life of its active metabolite is ∼40 h (Peng et al., 2005). Approximately 80% of an administered dose is excreted within 7 days (Peng et al., 2005), with >80% being excreted in the feces as metabolites or as IM by the action of ATP binding cassette (ABC) transporters (Gschwind et al., 2005). The specific PK parameters were exhibited in Table 1, which was supplied by a team from Switzerland in a single-center, open-label, randomized, crossover study (Nikolova et al., 2004).

**TABLE 1:** The pharmacokinetic parameters of IM and NDI following oral administration of 400 mg IM capsules or tablets according to one report.

Drug name	Formulation	t_1/2_ (h)	Cmax (ng/ml)	Tmax (h)	AUC_24_ (ng × h/mL)	CL/F (L/h)	References
IM	Tablet	15.9 ± 3.1	1,638 ± 604	2.5 (1.5–6.0)	19,019 ± 7,684	17.3 ± 5.1	Nikolova et al. (2004)
	Capsule	15.8 ± 2.9	1748 ± 702	2.5 (2.0–6.0)	19,959 ± 8,794	17.1 ± 5.8	
NDI	Tablet	39.9 ± 10.1	204 ± 89	2.5 (1.0–8.0)	2,457 ± 947	—	
	Capsule	37.4 ± 7.0	206 ± 80	2.5 (1.0–6.0)	2,563 ± 1,138	—	

IM, imatinib mesylate; NDI, *N*-desmethyl imatinib.

IM was formulated both as a tablet and as a capsule once, when it was first approved by United States-Food and Drug Administration (US-FDA); however, according to the information from US-FDA website, the capsule formulation was withdrawn or discontinued for safety- or efficacy-related reasons recently (United State Drug and Food Administration, 2001). Nowadays, the recommended dosage for adult patients with Philadelphia chromosome-positive CML at its various phases (chronic, accelerated, blast crisis) or KIT-positive unresectable and/or metastatic GISTs is 400 or 600 mg administered once daily with meals, as a monotherapy, according to the IM drug label.

Turn to the point, which we focused on, that is, the real outcome of mean plasma concentration of IM in the whole world. As shown in Table 2, we collected the data from IM-related clinic studies; the mean plasma concentration of IM was ranged from 800 to 1,500 ng/ml among 10 countries. The populations among 10 countries are Asian (China, Korea, Japan, and India), European (France, Norway, Netherlands, Belgium, and Italy), and North American (United States), respectively. The assays for measurement of plasma concentrations were based on liquid chromatography, high-performance liquid chromatography, and mass spectrometry. Studies conducted in different countries reported the mean plasma concentrations of IM in different formats and showed different effective values, indicating a lack of any uniform standard (Table 2).

**TABLE 2:** Steady-state plasma concentrations of imatinib mesylate with 400 mg/d dose.

Number	Mean concentrations (ng/ml)	Patients (numbers)	Assay	Country	References
1	1,521.26 ± 610.33	GIST, n = 168	HPLC	China	Wu et al. (2018)
2	1,398 ± 671	GIST, n = 209	LC-MS/MS	Korea	Koo et al. (2015)
3	904.8 ± 795.2	CML, n = 67	HPLC	Japan	Takahashi et al. (2010)
4	1,430.7 ± 438.7	CML, n = 111	LC-MS/MS	India	Francis et al. (2015)
5	868 ± 536	GIST, n = 96	LC-MS/MS	France	Bouchet et al. (2016)
6	1,332 ± 712	GISTs, n = 69	HPLC-MS	Norway	Hompland et al. (2016)
7	1,193 (from 227 to 4,606)	GISTs, n = 108	LC-MS	Netherlands	Farag et al. (2017)
8	1,099 ± 156	CML, n = 41	LC-MS/MS	Belgium	Van Obbergh et al. (2017)
9	800 (from 200 to 2,100)	CML, n = 24	HPLC-MS	Italy	De Francia et al. (2014)
10	The mean concentration was missing; but 71.9 and 66.6% of men and women reported mean concentration ranging from 1,000 to 2,000 ng/ml, respectively	CML, n = 93	HPLC-MS	United Kingdom	Belsey et al. (2017)

CML, chronic myeloid leukemia; GISTs, gastrointestinal stromal tumors; HPLC, high-performance liquid chromatography; LC, liquid chromatography; MS, mass spectrometry.

### Factors Affecting the Plasma Concentrations of Imatinib Mesylate

In this section, we focus on the factors affecting the plasma concentrations of IM in patients with GISTs or CML, respectively. Because the potency of NDI is similar to that of IM *in vitro*, the concentration of NDI is frequently determined along with IM concentrations in TDM studies (Zhuang et al., 2018).

#### Age

In the study of Bouchet’s team (Bouchet et al., 2013), over the period of January 2007 to October 2009, a total of 1985 blood samples were sent and used from French centers. Of these samples, the authors grouped the patients into three groups based on the age. The data exhibited that the mean C_min_ was 961 ng/ml, 1,034 ng/ml, and 1,134 ng/ml in the group of <45 years, 45–69 years, and >70 years, respectively. The differences were significant (*p* < 0.001) among these group. The concentration of IM tended to increase with the age, particularly in women. These findings indicate that elder patients would benefit the most from closer monitoring and early TDM-guided dose adjustments in case of warnings or adverse events. The potential mechanism inside the phenomenon might be related to a lower volume of distribution and elimination in older subjects (Larson et al., 2008). In this study, due to the elderly with higher IM mean C_min_ than younger group, the dosage adjusting in younger group bears further reflection.

#### Gender

Wu and coworkers (Wu et al., 2018) found significantly higher steady-state concentrations in females (1,680.79 ± 669.03 ng/ml, n = 86) than those in males (1,353.94 ± 492.89 ng/ml, n = 82; *p* < 0.01) after the administration of a 400 mg/d dose of IM. Another study (Koo et al., 2015) on 187 females and 122 males in Korea also demonstrated a significant correlation between the trough levels of IM with sex (*p* = 0.010). Although the authors did not supply the specific plasma concentration between male and female, the correlations between IM level and gender were analyzed by univariate linear regression, which also demonstrated female with higher IM level. A clinical study conducted in the United Kingdom (Belsey et al., 2017) examined 438 samples from 92 patients (53 males, 39 females) treated with a normalized dose of 400 mg/d. The mean plasma concentration of IM in female was 1.8 mg/L (n = 288, SD = 0.8), while it was 1.6 mg/L (n = 149, SD = 0.8) in male. No evidence was found of any association between gender and plasma IM levels (*p* = 0.13). However, the daily dose normalized mean plasma concentration of IM in female was 0.0043/L (n = 288, SD = 0.0018), while it was 0.0035/L (n = 149, SD = 0.0018) in male. This daily dose normalized mean plasma concentration was significantly higher in women in comparison with men (*p* < 0.05). Another outcome in this study was verifying the importance of measuring plasma NDI levels as NDI has pharmacological activity similar to that of IM. The mean plasma level and the daily dose normalized mean plasma level of NDI both exhibited significant differences between female and male CML patients (*p* < 0.05). Specifically, women were more likely to have higher NDI concentrations than men (*p* = 0.02). According to the explanation of authors, these results could be partly attributed to body weight, as women have a higher proportion of body fat. As IM could inhibit glycosaminoglycan synthesis on vascular proteoglycans and reduce low-density lipoproteins binding *in vitro* and *in vivo* via the platelet-derived growth factor receptor (Ballinger et al., 2010), the fat metabolism was close to the level of low-density lipoproteins (Dikariyanto et al., 2020).

#### Dosage

The dose level must be a force to be reckoned with affecting the therapeutic effect of IM in clinic. What’s more, the importance of monitoring for IM in clinic was because IM dose is a poorer predictor of drug exposure in the body. Through the analysis among the IM-related researches, there was only one study verifying the dosage of IM positive to the drug exposure. Bouchet et al. (2013) confirmed that C_min_ of IM was found to correlate with the reported IM dose, although the interpatient variability was high (∼60%). In this study, 1216 CML patients were recruited to examine the effects of different doses of IM: the dosages were classified as <400 mg (n = 230), 400 mg (n = 1,139), 600 mg (n = 480), and 800 mg (n = 61) daily. The mean C_min_ among these groups were 765, 944, 1,182, and 1,422 ng/ml, respectively.

A study from Korea (Yoo et al., 2010) aimed to evaluate the correlation between IM plasma concentration and clinical characterization in GISTs patients. They had recruited 107 patients who were taking IM 300–800 mg/d. In patients treated with IM 300 mg (n = 7), 400 mg (n = 92), 600 mg (n = 2), or 800 mg (n = 11) mg/d, IM C_min_ was 1,452 ± 830, 1,305 ± 633, 1,698 ± 725, and 3,330 ± 1,592 ng/ml, respectively. The significant difference was observed only between patients treated with 400 mg/d and those treated with 800 mg/d (*p* < 0.001).

In another report from China, groups of Zhang and colleagues (Zhang et al., 2018) conducted a phase IV, prospective, observational trial (registration number: ChiCTR-RNC-14004667) to explore the association of plasma IM concentrations with adverse drug reactions (ADRs) and the influence of genetic polymorphisms on ADRs in patients with GISTs treated with different doses of IM. Patients were treated with adjuvant IM at a dose of 400 mg/d, and patients harboring *KIT exon 9* mutations were initially treated with a dose of 600 mg/d. Patients who showed no response to 600 mg/d were switched to 800 mg/d of IM. If the patient was intolerant to IM or suffered from serious adverse events, the dose was reduced to 200–300 mg/d. In this clinical study, the mean steady-state plasma concentration of IM in 129 patients with GISTs was 1.45 ± 0.79 μg/ml. The plasma levels of patients treated with 600 mg/d of IM were significantly higher than those of other dosage groups (*p* < 0.05). The researchers presumed that the saturation effect of liver enzyme metabolism leads to a significant increase in the plasma concentration of IM in patients with 600 mg/day.

The outcome from Wu et al. (2018) for the effects of dosage on plasma concentrations demonstrated that IM was administered at doses of 300 mg/d (n = 16), 400 mg/d (n = 168), and >400 mg/d [500 mg/d (n = 1) and 600 mg/d (n = 5)] based on clinical diagnoses. The C_min_ levels were 1,564.5 ± 596.15 and 2,540.31 ± 1,298.14 ng/ml in patients treated with 300 mg/d and >400 mg/d of IM, respectively, which were significantly different from the C_min_ levels after a dose of 400 mg/d (*p* = 0.033).

The limitation of studies from Yoo et al. (2010), Zhang et al. (2018), and Wu et al., (2018) was insufficient sample capacity, which was 107, 129, and 190, respectively. Those aforementioned clinical outcomes also mirrored that dose of IM could not precisely predict the drug exposure in clinic. Because of these discrepancies in clinic, further investigation of the monitoring of IM is merited and recommended, with particular respect to the long-term and large sample. What’s more, it is extremely important to realize that some exploration of influential factors should be done after adjusting by dose level.

#### Gene Polymorphism

The genetic polymorphisms of the main drug-metabolizing enzymes and transporters may have a significant influence on interindividual variations in drug reaction and disposition (Uno et al., 2018). The time taken by tumors to develop resistance is usually different among patients undergoing IM therapy (Yoo et al., 2010). Such differences can be correlated with IM PK, which are closely related to genetic polymorphisms. The absorption, metabolism, transport, and clearance of IM depend on the efficiency of the proteins involved in these processes (Wasielewski et al., 2017).

IM is orally administered and is mainly metabolized by CYP enzymes and extruded by ABC subfamily B, member 1 (ABCB1), and ABC subfamily G, member 2 (ABCG2) (Pena et al., 2020). The nuclear receptor, pregnane x receptor (PXR, encoded by *NR1I2*), a member of the nuclear receptor superfamily, was found to simultaneously induce the expression of CYP3A4 and ABCB1 (Smutny et al., 2013). The organic cation transporter 1 (OCT) encoded by *SLC22A1* should not be neglected due to the ability for the uptake and elimination of endogenous small organic toxic byproducts and a large number of drugs (Jonker et al., 2003). The tumor is infiltrated by inflammatory cells. The role of inflammation plays an important factor in tumor growth, metastasis, or apoptosis Liu et al. (2015). Herein, the genetic polymorphism of inflammatory factor families, such as IL AND NR1l2 (Cheng et al., 2016), or inflammation closely related family, such as chemokine ligand (CXCL) family (Chen et al., 2020), gained the attention of the scientists.

As a result, those aforementioned genetic polymorphisms of IM were collected and described in Table 3. We focused on the outcomes, which not only displayed the significant correlation with the trough IM concentration, but also the differential results in the same single nucleotide polymorphism, because each of these results has to do with things that make sense for their problem domain. Specifically, some single nucleotide polymorphisms which demonstrated significant difference in the IM concentration deserved or called for more samples to verify whether it really should be taken into account before the IM administration or the dose adjustment. Some discrepancies or even opposite consequence should also be discussed for the potential reason, which could promote study design being more systemic and meaningful in the near future.

**TABLE 3:** Effect of gene polymorphism on the pharmacokinetics of IM in the regimen of GISTs or CML.

Number	Gene polymorphism	Dosage of IM	Sample and numbers	Major results	Country	References
1	CYP2C19 rs 28399505 (T>C)	400 mg/d	GISTs, n = 118	The patients’ plasma level with heterozygote TC was significantly lower than that with the wildtype TT.	China	Qiu et al. (2017)
2	CYP3A4 rs 2242480 (G>A)	400 mg/d	GISTs, n = 68	Patients with the GG genotype showed a significantly higher plasma level than patients with the GA + AA genotype	China	Liu et al. (2017)
3	CYP3A4 rs 755828176 (G>A)	400 mg/d	GISTs, n = 62	CYP3A4 (rs755828176) was significantly associated with unbound IM and NDI plasma trough levels	China	Qian et al. (2019)
4	CYP3A5-A6986G rs 776746	400 mg daily or twice daily	CML, n = 173	Patients with the GG genotype for CYP3A5-A6986G (*p* = 0.016) had significantly higher trough levels of IM.	India	Harivenkatesh et al. (2017)
5	ABCB1 rs1045642 (T>C)	400 mg/d	GISTs, n = 68	Patients with the TT + CT genotype showed significantly higher plasma levels than those with the CC genotype	China	Liu et al. (2017)
6	ABCB1 (1236T, 3435C>T, 2677G>T/A)	400 mg/d	CML, n = 90	Among patients who were homozygous for allele 1236T, 85% achieved a major molecular response vs. 47.7% for the other genotypes (*p* = 0.003). For the 2677 G>T/A polymorphism, the presence of the G allele was associated with a worse response. Patients with a 1236 TT genotype had higher IM concentrations. No association between C3435T polymorphism and response to ABVD was detected among HL patients (*p* > 0.05).	France	Mhaidat et al. (2011)
7	ABC1 3435C>T	400 mg/d	CML, n = 126	Patient with the TT and GG minor alleles of the SNPs, ABCB1, C3435T, and CYP3A5*3, showed lower clearance when compared with the CC and AA alleles, whereas the CT and AG had a higher clearance than the clearance of IM.	Nigeria	Adeagbo et al. (2017)
8	ABCB1 3435C>T	400 mg/d	CML, n = 73	Patients with this genotype showed no significant difference in the trough-level concentrations	India	Francis et al. (2015)
9	ABCB1 2677G>T/A	400 mg/d	CML, n = 73	Patients with this genotype showed no significant difference in the trough-level concentrations	India	Francis et al. (2015)
10	ABCG2 (421C>A)	400 mg/d	CML, n = 82	The CC, CA, and AA genotypes in ABCG2 421C>A yielded significantly different frequencies for MMR (*p* = 0.02); there were no statistically significant differences in the trough concentrations	Korea	Seong et al. (2013)
11	ABCG2 (421C>A)	400 mg/d	CML, n = 67	IM trough concentrations were significantly higher in the 25 patients with ABCG2 421C/A or 421A/A than in the 42 patients with 421C/C (*p* = 0.015)	Japan	Takahashi et al. (2010)
12	ABCG2 (421C>A)	400 mg/d	CML, n = 73	Patients with this genotype showed no significant difference in trough-level concentrations	India	Francis et al. (2015)
13	NR1I2 rs 3814055 (C>T)	400 mg/d	GISTs, n = 68	Patients with the CC genotype showed significantly higher plasma levels than those with the CT + TT genotype	China	Liu et al. (2017)
14	NR1I2 rs 3814055 (C>T)	400 mg/d	GISTs, n = 62	Patients with the CC genotype had significantly higher unbound IM dose-adjusted plasma trough concentrations (*p* = 0.040)	China	Qian et al. (2019)
15	IL13 rs1800925 and CXCL14 rs 7716492	400 mg/d	GISTs, n = 129	Mutations of IL13 rs1800925 and CXCL14 rs7716492 were related to the incidence of leukopenia and rash, separately (*p* < 0.05)	China	Zhang et al. (2018)
16	SLCO1A2 (-1105 G>A, -1032 G>A)	400 mg/d	CML, n = 34	IM clearance in patients with CML was influenced by the SLCO1A2 −1105g>a/−1032g>a genotype (*p* = 0.075) and the SLCO1A2 -361gg genotype	Japan	Yamakawa et al. (2011b)
17	SLCO1B3 (334T>G)	400 mg/d	CML, n = 34	Patients with genetic SLCO1B3 334T>G polymorphism had increased clearance rate of IM (*p* = 0.019)	Japan	Yamakawa et al. (2011a)
18	SLCO1B3 (334T>G)	400 mg/d	CML, n = 15	SLCO1B3 334T>G polymorphism could have had a significant impact on the intracellular concentration of IM in leukocytes, but it had no correlation with plasma concentrations	Japan	Nambu et al. (2011)
19	SLC22A1 (IVS6-878C>A, 1222A>G, IVS7+850C>T)	400 mg/d	CML, n = 38	This type of genotype was significantly associated with IM clearance	Singapore	Singh et al. (2012)
20	hOCT1 (c.480C4G)	400 mg/d	CML, n = 60	The c.480C4G genotype of hOCT1 had a significant effect on the apparent IM clearance	Italy	Di Paolo et al. (2014)
21	hOCT1 M408V (rs628031)	400 mg/d	CML, n = 106	M408V were significantly associated with the CCyR and MMR.	India	Vaidya et al. (2015)
22	OCT1 1386C>A	400 mg/d	CML, n = 73	Patients with this genotype showed no significant difference in the trough-level concentrations	India	Francis et al. (2015)
23	OCT1 1022C>T	400 mg/d	CML, n = 73	Patients with the genotype showed no significant difference in the trough-level concentrations	India	Francis et al. (2015)
24	OCT11222A>G	400 mg/d	CML, n = 73	Patients with this genotype showed no significant difference in the trough-level concentrations	India	Francis et al. (2015)

OCT, organic cation transporter 1; IM, Imatinib mesylate; CML, chronic myeloid leukemia.

As shown in Table 3, the single nucleotide polymorphism such as CYP2C19 rs28399505 (T>C), CYP3A4 rs2242480 (G>A), CYP3A4 rs755828176 (G>A), was exhibited significant correlation with IM plasma concentration. Taking CYP3A4 rs2242480 (G>A) as an example, the authors found it was not only a meaningful risk factor for predicting clinical efficacy of IM, but also related to ethnic population. Qiu et al. (2017) studied 118 patients with GISTs: 63 males, the remaining females, with a median age of 55 years. Patients received from 300 to 600 mg/d IM dose. The trough concentrations of IM in mutant allele A carriers (GA + AA) (n = 22, 2.27 ± 0.32 ng/mL/mg) were significantly lower than wildtype GG (n = 48, 4.12 ± 0.40 mg/mL/mg, *p* = 0.0171). The frequency of mutant allele A in this study was 17.58%, which was similar to healthy Han Chinese (22.1%) (Li et al., 2007), while significantly different from Caucasian (7.34%) and African (85.71%) (Wang et al., 2007). The mutant allele A was reported to be associated with a higher CYP3A4 metabolic activity (Ren et al., 2015), thus increasing the clearance of IM, which was mainly metabolized by CYP3A4, leading to lower IM plasma levels. Therefore, the mutant allele A of rs2242480 is a meaningful risk factor for predicting inadequate clinical efficacy of IM, and patients who carry mutant allele A of rs2242480 may be suggested to have a higher dose therapy. A positive correlation was confirmed in NR1I2 rs3814055 (C>T) from two Chinese studies in the patients with GISTs. In the report of Liu et al. (2015), patients with the CC genotype (n = 41, IM concentration = 4.26 ± 0.43 ng/mL/mg) showed significantly higher plasma levels than those with the CT + TT genotype (n = 27, IM concentration = 2.34 ± 0.25 ng/mL/mg, *p* = 0.0066). Similarly, patients with the CC genotype (n = 42, 0.0324 ± 0.0127 ng/mL/mg) had significantly higher unbound IM dose-adjusted plasma trough concentrations than TT genotype (n = 5, 0.0241 ± 0.0042 ng/mL/mg, *p* = 0.04) as demonstrated in the report of Qian et al. (2019).

Three groups with discrepant consequences raised our concern. There were gene polymorphisms in ABC1 3435 C>T, ABCG2 (421C>A), and SLCO1B3 (334T>G), respectively. Firstly, the clinical outcomes of the gene polymorphism in ABC1 3435 C>T were different among French, Nigerian, and Indian patient populations with the same disease and same therapeutic dosage. The reasons for discrepant consequences should have at least two sides. On the one side, the differences might be related to different population. France, Nigeria, and India belong to Europe, Africa, and Asia, respectively. The heredity and living environments were not similar. On the other side, the maximum sample size was 126 in those three studies, while the parameters were several genotypes, drug concentration, a series of serum index, and so one. A preliminary step before planning a clinical trial is the sample size calculation because the accuracy of consequence closely depends on the sufficient sample size (Miller et al., 2018). An important and often difficult step in sample size determination is to specify the required parameters for these methods (D’Arrigo et al., 2020). Here, we have to doubt whether there is a factor of too small sample size, causing the inconsistency of these abovementioned results.

Secondly, the results of the gene polymorphism in ABCG2 (421C>A) were inconsistent in three reports from Korea, Japan, and India in Table 3. The dosage and time of collection were the same in the Korean and Indian studies, whereas the time of collection differed in the Japanese study. The Japanese study collected data after 30 days of IM treatment, whereas the samples in the Korean study were collected after 6 months of daily IM therapy. The steady-state concentration was defined as 5–7 half-lives (t_1/2_) (Adeagbo et al., 2017). As the t_1/2_ of IM is ∼12 h in humans, this finding suggested that the steady state was reached in only 2.5–3.5 days. However, the best choice for monitoring is to constantly administer IM for at least 30 days depending on the clinical situation. Thus, the different clinical outcomes of the three studies from Korea, India, and Japan could be attributed to the different monitoring times. However, the differences in ethnicity of the three patient populations may also play a role in the clinical outcome after IM therapy. An ethnic group generally consists of a group of people who share a common heritage, similar social background, and culture. Ethnic differences in drug responses and efficacy have been found to be determined by genetic and environmental factors (Sai and Saito, 2011). Thus, these findings indicate that the ethnicity of the patient population should be considered carefully, especially in multicenter studies, to take into account any difference in clinical outcomes.

Thirdly, there was another interesting phenomenon reported by two sets of researchers in Japan for SLCO1B3 (334T>G). Yamakawa et al. (2011b) reported that SLCO1B3 (334T>G) increased the rate of clearance of IM in plasma (*p* = 0.019, n = 34), whereas Nambu et al. (Nambu et al., 2011) concluded that SLCO1B3 (334T>G) was related to the intracellular concentration and not the plasma concentration of IM. In both of these studies, the constant therapeutic period (IM was administered for more than 1 month), the monitoring assay (HPLC), the patients’ median age (about 50 years old), and the source of the patients (at Kumamoto University Hospital, Kumamoto, Japan) were identical. Only dosage ranges were different: one ranged from 100 to 400 mg/d; the other ranged from 100 to 600 mg/d. These results suggest that even minor differences may result in diverse clinical outcomes.

There was a common and obvious disadvantage of genetic polymorphism outcome in Table 3, which was the concern of insufficient sample capacity. To date, studies of large samples on the correlation between the IM plasma concentration and genetic polymorphism in patients with GISTs or CML are relatively rare. It is widely acknowledged that detection of plasma concentration of IM is of substantial guiding significance for medication instructions and dosage adjustment for GIST patients. Genetic polymorphisms have been always recognized as being either predictive or prognostic biomarkers, while the role of necessity was decreased by TDM in clinic. The reasons might need consideration from two sides. On the one hand, the rapid and intuitive outcome supplied by TDM could be more accessible. On the other hand, although the genetic polymorphisms had been studied increasingly, the results with inconsistent data or small sample size would not gain whole trust from clinicians.

#### Imatinib Mesylate-Drug Interactions

In clinical practice, combinations of drugs are administered because of the coexistence of different diseases. Administration of drugs along with IM in GIST therapy is common. As mentioned earlier, IM is metabolized predominantly in the liver by cytochromes CYP3A4 and CYP3A5 (Widmer et al., 2018), and it is mainly transported and excreted into bile by transporters related to P-glycoprotein (P-gp) (Maia et al., 2018). Hence, combining IM with a CYP inducer, CYP inhibitor, or a P-gp agonist would affect the plasma levels of IM.

The following PK parameters were used to assess the interactions between other drugs and IM (Smy et al., 2020): area under the concentration-time curve (AUC) from time zero to the last measurable sampling time point (AUC_(0–t)_), AUC from time zero to time infinity (AUC_(0–∞)_), apparent clearance (corrected for bioavailability) (CL/F), apparent volume of distribution at steady state, and elimination half-life (t_1/2_). Drug combinations used with IM therapy in the clinic are shown in Table 4 along with the above PK parameters.

**TABLE 4:** The results of drug combinations based on IM in GISTs or CML treatment regimens in the clinic.

Number	Role	Combinatorial drug	Dosage of IM	Sample and numbers	Major results	AUC	Apparent total plasma clearance	Apparent volume of distribution	Terminal elimination half-life	References
1	CYP inducer	Cyclophosphamide	400 mg/d	GISTs, n = 17	No drug-drug pharmacokinetic interactions were observed	The AUC _(0–24)_ of cyclophosphamide_ _and of IM were increased from 33.0 to 34.3 ng h/mL, which did not show any significant difference	—	—	The half-life of IM was increased from 14.1 to 16.4 h, which did not show significant difference	Adenis et al. (2013)
2	CYP inducer	Rifampicin	400 mg/d	Healthy people, n = 14	During concomitant rifampicin administration, the mean imatinib C_max_ decreased by 54%	The AUC _(0–24)_ and AUC_(0–∞)_ of IM decreased by 68 and 74%, respectively, during rifampicin treatment	The increase in clearance was 385% (348–426%) during rifampicin treatment	—	The half-life of IM decreased from 16.7 ± 3.1 to 8.8 ± 0.7 h during rifampicin treatment	Bolton et al. (2004)
3	CYP inducer	Phenytoin	400 –600 mg/d	CML, n = 1	Phenytoin could induce inadequate responses due to increased imatinib clearance and low IM trough plasma levels	In this case report, pharmacokinetic parameters were not supplied for IM or phenytoin. IM dose was increased from 400 to 600 mg and plasma trough level was increased to 458 ng/ml				Osorio et al. (2019)
4	CYP inhibitor	*Panax ginseng*	400 mg/d	CML, n = 1	A potential interaction resulting in liver toxicity between *P. ginseng* and IM.	In this case report, pharmacokinetic parameters were not supplied for IM or *P. ginseng*; however, the indices, such as alanine aminotransferase, aspartate aminotransferase, alkaline phosphatase, total bilirubin, albumin, and international normalized ratio, were described				Bilgi et al. (2010)
5	CYP inhibitor	Ketoconazole	200 mg/d	GISTs, n = 14	Ketoconazole increased the C_max_, AUC _(0–24),_ and AUC_(0–∞)_ by 26% (*p* < 0.005), 40% (*p* < 0.0005), and 40% (*p* < 0.0005), respectively	The AUC _(0–24),_ and AUC_(0–∞)_ of IM were increased from 9,618 ± 4,191 and 14,228 ± 7,359 to 13,498 ± 5,561 and 19,667 ± 8,932 ng h/ml, respectively, when treated with ketoconazole	The clearance of IM decreased from 16.3 ± 5.5 to 11.6 ± 4.0 L/h, when treated with ketoconazole	The apparent volume of distribution of IM did not show any significant difference, which varied from 472 ± 163 to 318 ± 113 L, when treated with ketoconazole	The half-life of IM did no show significant difference (from 20.5 ± 4.4 to 19.2 ± 4.5 h), when treated with ketoconazole	Dutreix et al. (2004)
6	P-gp substrate	Cyclosporine A	400 or 600 mg/d	CML with underwent hematopoietic stem cell transplantation, n = 16	Based on measured drug concentrations, the cyclosporine dosage needed to be reduced, on average, by 27% after initiation of IM (*p* = 0.004)	The pharmacokinetic parameters were not supplied for IM or cyclosporine a. The authors measured the plasma concentration of cyclosporine a only				Atiq et al. (2016)
7	P-gp agonists	St. John’s wort	400 mg/d	Healthy people, n = 12	A 43% increase in clearance and a 30% decrease in the AUC of IM were observed	The AUC _(0–24)_ of IM was decreased by 30%, when treated with st. John’s wort	The clearance of IM decreased from 17.9 to 12.5 L/h, when treated with st. John’s wort	—	The half-life of IM decreased from 12.8 to 9.0 h, when treated with st. John’s wort	Frye et al. (2004)
8	Cytokine	Interleukin-2	400 mg/d	GISTs, n = 21	IL-2 increased the maximum concentration of IM and its main metabolite NDI; IM did not modulate IL-2 pharmacokinetics	The AUC _(0–24)_ and AUC_(0–∞)_ of IM were increased from 39.6 ± 13.4 and 64.1 ± 28.2 to 64 ± 16.5 and 94.6 ± 30.6 μg h/mL, respectively, when treated with interleukin-2	The clearance of IM decreased from 7.8 ± 4.9 to 4.7 ± 1.7 L/h, when treated with interleukin-2	The apparent volume of distribution of IM decreased from 126.5 ± 64.2 to 82.9 ± 24.5 L, when treated with interleukin-2.	The half-life of IM did not show significant difference (from 13.1 ± 8.9 to 13 ± 3.9 h), when treated with interleukin-2	Pautier et al. (2013)
9	Cyclin-dependent kinase inhibitor	Flavopiridol	400 mg/d	CML, n = 21 (17 patients had pharmacokinetic data for IM.)	The combination of flavopiridol and IM is tolerable and produces encouraging responses	AUC values of IM were on average 21% lower in the presence of flavopiridol (*p* = 0.022) vs. IM’s metabolite, NDI (*p* = 0.84)	In comparing concentrations immediately prior to and at the end of the 1 h administration of flavopiridol, flavopiridol increased the concentrations of NDI (*p* = 0.007) but did not affect IM plasma concentrations (*p* = 0.44). Although the changes in NDI concentrations were statistically significant, the magnitude of these effects was considered to be small			Bose et al. (2012)
10	CYP substrate	Gefitinib	400 mg/d	CML with lung cancer, n = 1	Liver function and pancreatic enzyme values gradually worsened after initiation of IM, and the patient was diagnosed with acute pancreatitis. IM was discontinued owing to pancreatic toxicity after 22 days. Lastly, the patient was readmitted with significant deterioration of her clinical situation and died 6 days later	There were no IM or gefitinib plasma concentration data in this case report, only potential discussion from the authors. The interaction most likely arose because imatinib is a CYP2D6 inhibitor and could therefore impair the metabolism of gefitinib (a CYP2D6 substrate) and increase its serum concentration as in the authors analysis				(Escudero-Vilaplana et al. (2020)
11	CYP substrate	Sertraline	400 mg/d	CML, n = 1	Final diagnosis was subfulminant toxic acute hepatitis secondary to IM and sertraline treatment	The Naranjo nomogram showed a probable correlation between this adverse effect and the interaction between IM and sertraline. This interaction is extremely rare and the mechanism of action is not clear				Strawn et al. (2019)

CML, chronic myeloid leukemia; IM, imatinib mesylate; GISTs, gastrointestinal stromal tumors; AUC_0–24_ = area under the serum concentration-time curve from time zero extrapolated to 24 h; AUC_0–∞_ = area under the serum concentration-time curve from time zero extrapolated to infinity.

As exhibited in Table 4, there were 4 case reports about IM-drug interaction, which contained phenytoin (a CYP inducer) (Osorio et al., 2019), *Panax ginseng* [a CYP inhibitor (Bilgi et al., 2010)], gefitinib [a CYP substrate (Escudero-Vilaplana et al., 2020)] and sertraline [a CYP substrate (Strawn et al., 2019)]. Among these four cases, only conclusion from case of phenytoin showed that it could induce inadequate responses due to increased IM clearance and low IM trough plasma levels. The case report of *Panax ginseng* supplied some liver function indices, such as alanine aminotransferase, aspartate aminotransferase, alkaline phosphatase, total bilirubin, albumin, and international normalized ratio to infer the interaction between IM and *Panax ginseng,* while in the case of the gefitinib and sertraline with the help of Naranjo nomogram assay (Naranjo et al., 1981) the drug interaction was verified. Herein, we would take gefitinib as an example to introduce this drug interaction. In combination with other anticancer drugs in the patients with GISTs or CML is a situation that has to be faced clinically. Concomitant use of various TKIs for the treatment of different tumors in the same patient is very rare, and there are no published data on their safety or potential drug interactions. Here, we presented the case of a patient who received the TKIs IM and gefitinib for the treatment of CML and lung adenocarcinoma, respectively, from a report of Escudero-Vilaplana et al. (2020). A woman was diagnosed with CML and, then after 9 years, with stage IV lung cancer. The patient continued treatment with gefitinib 250 mg daily and IM 400 mg daily. After 22 days of cotreatment, the patient progressed worsening of liver function and pancreatic enzyme values. The Naranjo nomogram assay (Naranjo et al., 1981) was chosen to evaluate the relationship between adverse reaction (ADR) and drug interaction. The ADR was assigned to a probability category from the total score as follows: definite ≥9, probable 5 to 8, possible 1 to 4, and doubtful ≤0 (Naranjo et al., 1981). In this case report, the score of Naranjo nomogram was 4, which indicated a possible correlation between this adverse effect and drug interaction. Herein, the IM was discontinued. In the researchers’ analysis, the interaction potential indicated that IM is a CYP2D6 inhibitor and could therefore impair the metabolism of gefitinib (a CYP2D6 substrate) and increase its serum concentration. The limitation in this case was that the specific plasma concentration of IM or gefitinib was not monitored, which could not directly reflect the relationship between drug concentration and drug interaction. Also, the concurrent use of IM with sertraline, an antidepressant drug, in the treatment of CML brought acute hepatitis in a case report (Strawn et al., 2019). The Naranjo nomogram showed a probable correlation between this adverse effect and the interaction between IM and sertraline (score = 7), while the actual plasma concentration of IM or sertraline was missing too.

Besides those case reports, there were seven researches about IM-drug interaction design, while the maximum number of included samples was only 21. The PK parameters in the research of ketoconazole (a CYP inhibitor) and interleukin-2 (a cytokine) were complete, while in the remaining five studies they were incomplete. Hence, we shall present the study of ketoconazole as an example. Patients with GISTs and HIV together may need antiviral therapy and IM simultaneously. Beumer and coworkers (Beumer et al., 2015) used LC-MS assay to investigate the concentrations of IM in human liver sample. The included samples were from patients with GISTs or CML and HIV at the same time. The researchers found that the clearance of IM was 4.0-fold lower, 2.8-fold lower, 2.9-fold higher, and 2.0-fold higher after its oral administration along with ketoconazole, ritonavir, rifampicin, and efavirenz, respectively. These findings suggested that TDM might be extremely critical in the setting of antiretroviral comedication with IM in clinic.

Another study has to be mentioned, in which there was a drug interaction between IM and cyclophosphamide. Cyclophosphamide is a known CYP inducer, which decreases the C_min_ of IM in a mouse model (Chun et al., 2014). However, no drug-drug PK interactions were observed in patients with GISTs when cyclophosphamide was administered along with IM. These different outcomes could be attributed to the differences between humans and rats, especially between patients with GISTs and normal rats. A basic research had verified that the adenine dinucleotide phosphate (ADP+) adducts of IM after incubations with rat and human liver microsomes exhibit different phenomena (Ma et al., 2008). Specifically, only trace levels of ADP+ adducts of IM and NDI were detected in the incubations with human liver microsomes. Even though the role of ADP+ adducts was not clear in rat or human, the result in this study had proved that the rat model and the human model should be different in the metabolism with IM.

Based on our literature review, we found that insufficient attention has been directed to drug interactions with IM in GISTs or CML patients. Indeed, there is not much information on the PK parameters altered by drug combinations with IM that could help in investigating the actual drug interactions. Furthermore, the sample size included was very scanty. Lastly, the analysis assay about drug interaction was nonuniform and not systemic.

#### Pathology

Pathology, especially after gastric surgery in patients with GISTs, could dramatically affect the IM concentration in plasma. After a dose of 400 mg/d, C_min_ of IM was significantly higher in Chinese patients who had not undergone gastrectomy (1,649.88 ± 620.12 ng/ml, n = 69) than in patients who had undergone gastrectomy (1,439.60 ± 587.66 ng/ml, n = 84, *p* = 0.033) (Wu et al., 2018). Similarly, in Korea, Yoo et al. (2010) reported that C_min_ was significantly lower in patients who had previously undergone major gastrectomy (942 ± 330 ng/ml, n = 18) than in those with previous wedge gastric resection or without gastric surgery (1,393 ± 659 ng/ml, n = 74, *p* = 0.002). Furthermore, multivariate analysis revealed that the concentration of albumin (*p* = 0.001), creatinine clearance (*p* = 0.002), and major gastrectomy (*p* = 0.003) were significantly correlated with the steady-state concentration of IM. The lower C_min_ observed in patients with gastric resection has been attributed to the lack of gastric acid secretion. As IM tablets dissolve rapidly at a pH of 5.5 or less, the absorption of IM decreases partly under these conditions. These findings indicate that despite the lack of clarity about the exact mechanism, clinicians should pay more attention to surgery as it may affect the absorption of IM.

When the gastric surgery was confirmed correlative with IM plasma concentration by some scientists, the localization of GISTs was included to investigate. A retrospective study conducted in Norway (Bouchet et al., 2016) included 96 GISTs patient to participate in this study. The reported localizations of GISTs were stomach (n = 41), small bowel (n = 34), colorectal (n = 8), and other (n = 13): esophageal (n = 1), mesenteric (n = 4), rectovaginal (n = 2), anal (n = 1), pelvic (n = 1), and difficult to locate (n = 4). Although the inclusion localization of GISTs was diverse, the authors just compared the IM concentration in the group of stomach with small bowel for some reasons. For those with stomach GISTs localization mean C_min_ was 793 ± 535 ng/ml and for those with small bowel localization C_min_ was 998 ± 623 ng/ml, which did not differ statistically. The progression rate was 3-fold higher for small bowel than stomach in the GISTs patients (38 vs. 12%) with the help of Kaplane-Meier statistical analysis. The pity of this study was that the influence of different localization in GISTs did not clarify clearly, especially without the specific data of the plasma concentration of the pathological locations of colorectal, esophageal, mesenteric, and rectovaginal origin, and so on. This study might point out a new direction for exploring the affection of pathology on IM plasma concentration and further effect.

Gomez-Samano et al. (2018) from Mexico reported that patients with CML or GISTs with type 2 diabetes mellitus had a simultaneous, clinically significant reduction in mean fasting plasma glucose and HbA1c levels at 1 month and 6 months of IM therapy. The potential mechanism demonstrated by the authors was that IM inhibited the phosphorylation of proteins which may result in signaling with improvement in insulin sensitivity. Thus, HbA1c level was decreased. However, the plasma concentrations of IM were not measured in these subjects. Based on these results, clinicians should consider the hypoglycemic effect of IM when treating the aforementioned diseases.

#### Food

IM as an oral administration drug is not free from problems such as influence of food. Considering the literature searching, there is no systemic study about specific food affecting the IM. The international BFM Group Study Group Chronic Myeloid Leukemia Committee recommended that IM is ought to be taken in a sitting position with a large glass of water or apple juice (minimum 100 ml) for the management of CML in children and young people up to the age of 18 years owing to the local irritation of IM (de la Fuente et al., 2014). According to the label of IM, the solubility of IM in aqueous buffers of pH ≤ 5.5, the acidic pH of apple juice (pH 3.5) is considered to be advantageous to facilitate the absorption of IM (Collado-Borrell et al., 2016).

Herein, the influence of food should be indicated as a research interest to take into account.

#### Other Factors

Some unconventional factors, such as smoking and compliance, could be broadening our horizons to realize the drug function. Van Erp et al. (2008) explored the effects of smoking on the concentration of IM in patients with GISTs. They did not find any significant differences in the AUC 133.6 ± 71.0 versus 142.3 ± 84.0 ng h/mL mg in smokers (n = 9) versus nonsmokers (n = 25), respectively.

The half-life of IM is in the region of 18 h; thus lack of compliance for just 1 week would completely eliminate the drug from plasma (Marin et al., 2010). Several recent studies have shown that lack of adherence to IM has a significant impact on the degree of response (Marin et al., 2010; Ibrahim et al., 2011), while these studies focused on the relationship between CCyR and adherence ignoring the IM plasma concentration with adherence, especially in the condition that IM was reused after 1 week of discontinuation.

The acute-phase protein, alpha-1 acid glycoprotein (AGP), played essential role in bounding with IM. The reduction in AGP would lead to less protein-bound IM and increase of free IM that could be metabolized or excreted (Eechoute et al., 2012). According to this theory, it is assumed that AGP levels should be closely correlated with IM. *Sander* Bins et al. (2017) made a prospective setting that measured IM trough concentration with AGP level synchronously. Totally 69 GISTs patients were included and three time points were set (30, 60, and 365 days after IM treatment) in this study. When IM trough concentrations were 1,457 (1,155–1,838) ng/ml, 1,305 (1,001–1702) ng/ml, and 1,193 (967–1,472) ng/ml, the AGP levels were 0.97 (0.85–1.10)g/L, 0.81 (0.69–0.94) g/L, and 0.89 (0.78–1.00) g/L, respectively. Correlation was tested using Pearson’s correlation and *p* value, which were depicted as *r* ^2^ and *p*. The results demonstrated that concentration of IM was closely correlated with AGP levels when all samples were considered together (*r* ^2^ = 0.656, *p* = 0.001).

## Conclusion

Plasma IM concentrations exhibit high interindividual and intraindividual variability. Alterations in the PK of IM may result in undesirable outcomes and require close clinical monitoring. In this review, we found something worth improving to study the factors influencing the steady-state plasma concentration of IM, which was summarized as below.

1) Steady-state plasma concentration patients did not have the same inclusion criteria. Some had been on monotherapy with 400 mg of IM per day for at least 30 days (Adeagbo et al., 2017; Farag et al., 2017; Van Obbergh et al., 2017) and had taken the drug consistently for the last 5 days prior to the time of blood collection; others were on the therapy for at least 90 days (Hompland et al., 2016) or even 120 days (Seong et al., 2013). Whether IM resistance or disease progression will occur with the extension of IM treatment time might be a potential factor to affect the IM trough concentration. Thus, based on the findings reported in this review, we call for a consensus guideline for monitoring IM, which should stipulate a fixed blood collection time and a standard format for reporting mean concentrations of IM in clinical studies.
2) Based on all the studies covered in this article, we found that although the effective therapeutic plasma concentrations of IM in patients with GISTs and CML received attention at levels above 1,000 or 1,100 ng/ml, there was no information about the critical value of IM in plasma. The critical value of IM plasma concentration being missing might be caused by two parts. On the one side, it is rare to use IM beyond the prescribed dosage. On the other side, IM was relatively well tolerated. Herein, whether the critical value of IM in plasma could be firstly studied in animal model was a question worth thinking about.
3) Gene polymorphisms were examined to determine their effect on IM therapy. These different gene polymorphisms might be related to ADRs, MMRs, and racial differences and so on. In order to increase the clinic recognition and value of gene polymorphisms, the more sample capacity, multicenter, long-term study was worthy to carry out. What’s more, gene polymorphisms deserve more attention, especially in combination therapy, and the relationship between gene polymorphisms and plasma IM concentration must be investigated with the help of specific PK parameters.
4) Finally, drug-drug interactions are common with IM, regardless of whether they occur in patients with GISTs or CML. Hence, it is important to assess concurrent medications, especially CYP inhibitors, CYP inducers, Pgp inhibitors, or PgP inducers, for drug-drug interactions with IM. The experimental design for drug interaction with IM needed the PK parameter for both including drug and drug interaction analyzing assay, such as Naranjo nomogram assay, instead of conducting it only by some clinical serum index.

## Author Contributions

YC conceived and wrote the paper; XhD revised the edition for revising the language, adjusting the framework, updating corresponding references; QW revised and edited the paper; ZL and XwD collected data from references; and SS and HX aided in conceptualization and the supporting funding.

## Funding

This work was supported by the Healthy Department of Sichuan Province (Grant No. 20PJ110; 20PJ116; Cadre Health Care Research Project of Sichuan (Grant No. 2019-801); Science and Technology Program of Sichuan Province (Grant No. 2020JDTD0029, 2020YFS0412); Sichuan Cancer Hospital & Institute independently funded the project (Grant No. YB2019001) and Sichuan Provincial Key Discipline of Medicine (Pharmacy).

## Conflict of Interest

The authors declare that the research was conducted in the absence of any commercial or financial relationships that could be construed as a potential conflict of interest.
